# Estimation of SARS-CoV-2 Neutralizing Activity and Protective Immunity in Different Vaccine Types Using Three Surrogate Virus Neutralization Test Assays and Two Semiquantitative Binding Assays Targeting the Receptor-Binding Domain

**DOI:** 10.1128/spectrum.02669-22

**Published:** 2022-10-17

**Authors:** Beomki Lee, Jae-Hoon Ko, Kyoung Hwa Lee, Yong Chan Kim, Young Goo Song, Yoon Soo Park, Yae Jee Baek, Jin Young Ahn, Jun Yong Choi, Kyoung-Ho Song, Eu Suk Kim, Seongman Bae, Sung-Han Kim, Hye Won Jeong, Shin-Woo Kim, Ki Tae Kwon, Su-Hwan Kim, Hyeonji Jeong, Byoungguk Kim, Sung Soon Kim, Won Suk Choi, Kyong Ran Peck, Eun-Suk Kang

**Affiliations:** a Department of Laboratory Medicine and Genetics, Samsung Medical Center, Sungkyunkwan University School of Medicine, Seoul, South Korea; b Division of Infectious Diseases, Department of Medicine, Samsung Medical Center, Sungkyunkwan University School of Medicine, Seoul, South Korea; c Division of Infectious Diseases, Department of Internal Medicine, Gangnam Severance Hospital, Yonsei University College of Medicine, Seoul, South Korea; d Division of Infectious Diseases, Department of Internal Medicine, Yongin Severance Hospital, Yonsei University College of Medicine, Yongin, South Korea; e Department of Internal Medicine, Severance Hospital, Yonsei University College of Medicine, Seoul, South Korea; f Department of Internal Medicine, Seoul National University Bundang Hospital, Seoul National University College of Medicine, Seongnam, South Korea; g Department of Infectious Diseases, Asan Medical Center, University of Ulsan College of Medicine, Seoul, South Korea; h Department of Internal Medicine, Chungbuk National University College of Medicine, Cheongju, South Korea; i Department of Internal Medicine, School of Medicine, Kyungpook National University, Daegu, South Korea; j Division of Infectious Diseases, Department of Internal Medicine, Kyungpook National University Chilgok Hospital, School of Medicine, Kyungpook National University, Daegu, South Korea; k Korea Disease Control and Prevention Agency, Cheongju, Chungcheongbuk, South Korea; l Division of Infectious Diseases, Department of Internal Medicine, Ansan Hospital, Korea University College of Medicine, Ansan, South Korea; Johns Hopkins Hospital

**Keywords:** SARS-CoV-2, COVID-19, neutralization tests, sVNT, ChAdOx1, BNT162b2, binding assays

## Abstract

Estimating neutralizing activity in vaccinees is crucial for predicting the protective effect against severe acute respiratory syndrome coronavirus 2 (SARS-CoV-2). As the plaque reduction neutralization test (PRNT) requires a biosafety level 3 facility, it would be advantageous if surrogate virus neutralization test (sVNT) assays and binding assays could predict neutralizing activity. Here, five different assays were evaluated with respect to the PRNT in vaccinees: three sVNT assays from GenScript, Boditech Med, and SD Biosensor and two semiquantitative binding assays from Roche and Abbott. The vaccinees were subjected to three vaccination protocols: homologous ChAdOx1, homologous BNT162b2, and heterologous administration. The ability to predict a 50% neutralizing dose (ND_50_) of ≥20 largely varied among the assays, with the binding assays showing substantial agreement (kappa, ~0.90) and the sVNT assays showing relatively poor performance, especially in the ChAdOx1 group (kappa, 0.33 to 0.97). The ability to predict an ND_50_ value of ≥118.25, indicating a protective effect, was comparable among different assays. Applying optimal cutoffs based on Youden’s index, the kappa agreements were greater than 0.60 for all assays in the total group. Overall, relatively poor performance was demonstrated in the ChAdOx1 group, owing to low antibody titers. Although there were intra-assay differences related to the vaccination protocols, as well as interassay differences, all assays demonstrated fair performance in predicting the protective effect using the new cutoffs. This study demonstrates the need for a different cutoff for each assay to appropriately determine a higher neutralizing titer and suggests the clinical feasibility of using various assays for estimation of the protective effect.

**IMPORTANCE** The coronavirus disease 2019 (COVID-19) pandemic continues to last, despite high COVID-19 vaccination rates. As many people experience breakthrough infection after prior infection and/or vaccination, estimating the neutralization activity and predicting the protective effect are major issues of concern. However, since standard neutralization tests are not available in most clinical laboratories, it would be beneficial if commercial assays could predict these aspects. In this study, we evaluated the performance of three sVNT assays and two semiquantitative binding assays targeting the receptor-binding domain with respect to the PRNT. Our results suggest that these assays could be used for predicting the protective effect by adjusting the cutoffs.

## INTRODUCTION

Severe acute respiratory syndrome coronavirus 2 (SARS-CoV-2) has caused a lengthy pandemic globally, with the emergence of variants with different mutations. While the vaccination rate has reached 85.4% and the booster vaccination rate 49.2% in Korea as of January 2022 (1), breakthrough infections are still a major concern. As of 20 March 2021, there were 90,804 domestic patients with coronavirus disease 2019 (COVID-19), with a first-dose vaccination rate of 84.4% in Korea (2). Recent studies have demonstrated that a booster shot was successful in diminishing the rate of infection and severity of COVID-19 (3–5). In line with this, booster vaccinations are actively administered after a second primary dose of the Oxford-AstraZeneca ChAdOx1 novel coronavirus 2019 (nCoV-19) and Pfizer-BioNTech BNT162b2 vaccines and after a single dose of the Johnson & Johnson-Janssen Ad26.COV2.S vaccine in Korea (6–8). To predict vaccine efficacy, estimation of the neutralization capacity of anti-SARS-CoV-2 spike protein antibodies is critical.

As the standard methods for measuring the neutralization titer, such as the plaque reduction neutralization test (PRNT), use live pathogens, a biosafety level 3 (BSL3) facility is required, which is usually not feasible in clinical laboratories. In addition, the low throughput, long turnaround time, and suboptimal standardization of the PRNT are factors that make it inappropriate for clinical laboratories. To overcome these limitations, a method called the surrogate virus neutralization test (sVNT) has been developed that can be performed in BSL2 clinical laboratories (9). The sVNT determines the inhibition rate by measuring the degree of interaction between the human angiotensin-converting enzyme 2 receptor and the SARS-CoV-2 spike protein receptor-binding domain (RBD) (10). In addition to the widely known cPass SARS-CoV-2 neutralization antibody detection kit (GenScript, Piscataway, NJ, USA) (GS), sVNT assays utilizing similar principles have been developed in several countries, including Korea. Beyond sVNT assays, while semiquantitative binding assays do not measure the neutralization effect *per se*, those targeting the RBD of the spike protein have shown a correlation with the neutralization titer in patients with COVID-19 (11).

In this study, we aimed to evaluate the capability of various assays to estimate the neutralization activity compared to the PRNT in vaccinees subjected to different vaccine protocols and to address the titer correlation with respect to the PRNT.

## RESULTS

### Qualitative evaluation.

While the semiquantitative binding assays showed almost perfect agreement with a PRNT 50% neutralizing dose (ND_50_) of ≥20, the sVNT assays showed relatively poor agreement. Among the three sVNT assays, qualitative agreement with the PRNT ND_50_ value of ≥20 was the highest for GS regardless of the vaccine type (kappa, 0.69 to 0.97). When the different vaccine types were compared, the BNT162b2 group showed a significantly higher agreement (kappa, 0.76 to 0.99) in all assays than the other two groups. While all assays demonstrated high specificities (87.59 to 100.00%), the sensitivities varied by a significant magnitude across the different assays and vaccine types (46.52 to 99.67%). The sensitivities, specificities, and kappa agreements are listed in Table 1.

**TABLE 1:** Performance of various assays using an ND_50_ value of ≥20 as the PRNT cutoff

Assay (cutoff)	Vaccine protocol	Performance		
		Sensitivity (95% CI)^*a*^	Specificity (95% CI)	Kappa
GenScript (30%)	Total	89.19 (86.85–91.24)	97.57 (94.79–99.10)	0.78
	ChAdOx1	81.52 (76.90–85.56)	99.24 (95.85–99.98)	0.71
	BNT162b2	98.33 (96.14–99.45)	100.0 (96.41–100.0)	0.97
	Heterologous^*b*^	80.38 (75.57–84.61)	95.86 (91.21–98.47)	0.69
Boditech Med (30%)	Total	75.65 (72.54–78.56)	93.93 (90.18–96.56)	0.55
	ChAdOx1	60.30 (54.80–65.62)	94.70 (89.38–97.84)	0.43
	BNT162b2	90.97 (87.13–93.96)	96.04 (90.17–98.91)	0.81
	Heterologous	62.54 (56.94–67.90)	92.41 (86.83–96.15)	0.46
SD Biosensor (20%)	Total	67.81 (64.48–71.01)	97.17 (94.25–98.85)	0.48
	ChAdOx1	55.76 (50.22–61.19)	96.21 (91.38–98.76)	0.39
	BNT162b2	87.29 (82.98–90.85)	98.02 (93.03–99.76)	0.76
	Heterologous	46.52 (40.92–52.19)	96.55 (92.14–98.87)	0.33
Roche (0.82 BAU/mL)	Total	98.53 (97.44–99.24)	93.52 (89.69–96.25)	0.93
	ChAdOx1	96.67 (94.11–98.32)	99.24 (95.85–99.98)	0.94
	BNT162b2	99.67 (98.15–99.99)	99.01 (94.61–99.97)	0.99
	Heterologous	96.52 (93.86–98.25)	89.66 (83.51–94.09)	0.87
Abbott (7.1 BAU/mL)	Total	97.79 (96.53–98.68)	91.09 (86.83–94.33)	0.89
	ChAdOx1	95.15 (92.25–97.20)	96.21 (91.38–98.76)	0.89
	BNT162b2	99.67 (98.15–99.99)	96.04 (90.17–98.91)	0.97
	Heterologous	95.25 (92.29–97.32)	87.59 (81.09–92.47)	0.83

a CI, confidence interval.

b Since the heterologous group was enrolled after the second sample collection of the ChAdOx1 and BNT162b2 groups, the results of the first and second sample collections of the ChAdOx1 group were imputed.

**FIG 1:**
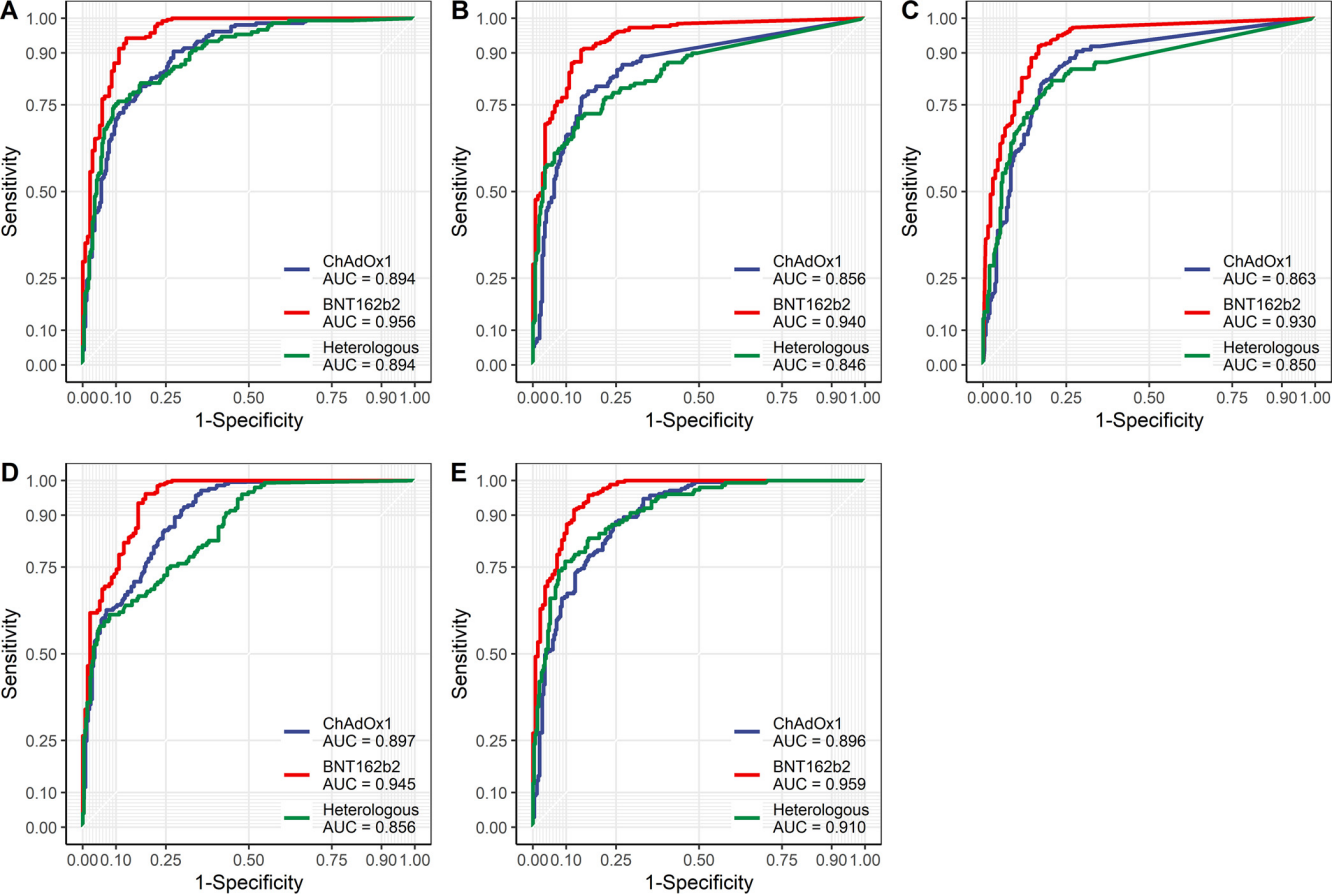
Receiver operating characteristic (ROC) curve of each assay using an PRNT ND_50_ value of ≥118.25: (A) GenScript, (B) Boditech Med, (C) SD Biosensor, (D) Roche, and (E) Abbott.

To estimate the protective effect using commercial assays, the performance of each assay with respect to a PRNT ND_50_ value of ≥118.25 was evaluated (Table 2). When the manufacturer cutoffs were applied, the GS, Roche, and Abbott assays showed lower agreement with the PRNT than for the evaluation using a PRNT ND_50_ value of ≥20. On the contrary, the Boditech Med (BM) and SD Biosensor (SD) assays showed better performance in predicting the protective effect in the ChAdOx1 and heterologous groups. When the receiver operating characteristic (ROC) curve for a PRNT ND_50_ value of ≥118.25 was implemented for each assay and vaccine protocol (Fig. 1), all assays showed comparable results, with area under the curve (AUC) values of at least 0.846, suggesting that the manufacturer cutoffs are not suitable for predicting the protective effect using an PRNT ND_50_ value of ≥118.25. To overcome this drawback, different cutoff values representing the protective effect were obtained for each assay using Youden’s index: GS, 53.05%; BM, 48%; SD, 15%; Roche, 278.81 binding antibody units (BAU)/mL; and Abbott, 57.48 BAU/mL. The GS, Roche, and Abbott assays showed superior performance in predicting the protective effect using the newly determined cutoffs than that achieved using the manufacturer cutoffs. As the new cutoffs were higher than the manufacturer cutoffs, higher agreement was achieved with higher specificity (77.54 to 94.86%), sacrificing sensitivity (58.00 to 95.42%).

**TABLE 2:** Performance of various assays with a PRNT ND_50_ value of ≥118.25 using the manufacturer cutoffs and the cutoffs determined using Youden’s index

Cutoff determination	Assay (cutoff)	Vaccine protocol	Performance		
			Sensitivity (95% CI)	Specificity (95% CI)	Kappa
Manufacturer	GenScript (30%)	Total	96.27 (94.35–97.68)	61.85 (57.42–66.13)	0.59
		ChAdOx1	91.39 (86.73–94.82)	68.77 (62.67–74.43)	0.59
		BNT162b2	99.24 (97.27–99.91)	75.36 (67.31–82.29)	0.79
		Heterologous^*a*^	93.33 (88.08–96.76)	61.41 (55.76–66.85)	0.46
	Boditech Med (30%)	Total	87.54 (84.53–90.16)	72.29 (68.13–76.18)	0.60
		ChAdOx1	78.47 (72.27–83.84)	83.40 (78.23–87.77)	0.62
		BNT162b2	93.51 (89.81–96.18)	77.54 (69.66–84.20)	0.73
		Heterologous	79.87 (72.52–85.98)	71.38 (66.01–76.34)	0.46
	SD Biosensor (20%)	Total	84.01 (80.72–86.95)	82.73 (79.12–85.95)	0.67
		ChAdOx1	72.73 (66.15–78.64)	85.38 (80.41–89.49)	0.59
		BNT162b2	91.60 (87.56–94.66)	83.33 (76.05–89.13)	0.75
		Heterologous	72.67 (64.80–79.62)	86.17 (81.83–89.81)	0.59
	Roche (0.82 BAU/mL)	Total	99.82 (99.01–100.0)	48.59 (44.12–53.08)	0.50
		ChAdOx1	99.52 (97.36–99.99)	55.73 (49.38–61.95)	0.53
		BNT162b2	100.0 (98.60–100.0)	73.19 (64.99–80.37)	0.78
		Heterologous	99.33 (96.34–99.98)	45.02 (39.40–50.73)	0.34
	Abbott (7.1 BAU/mL)	Total	99.11 (97.94–99.71)	47.79 (43.33–52.28)	0.48
		ChAdOx1	97.61 (94.51–99.22)	54.55 (48.19–60.79)	0.50
		BNT162b2	100.0 (98.60–100.0)	71.01 (62.69–78.42)	0.76
		Heterologous	98.00 (94.27–99.59)	44.69 (39.08–50.41)	0.33
Youden’s index	GenScript (53.05%)	Total	84.90 (81.67–87.76)	85.54 (82.14–88.51)	0.70
		ChAdOx1	70.81 (64.14–76.88)	90.12 (85.76–93.50)	0.62
		BNT162b2	92.75 (88.91–95.58)	87.68 (81.01–92.66)	0.80
		Heterologous	78.00 (70.51–84.35)	85.85 (81.48–89.53)	0.63
	Boditech Med (48%)	Total	79.36 (75.77–82.63)	85.14 (81.71–88.15)	0.64
		ChAdOx1	66.03 (59.18–72.42)	90.12 (85.76–93.50)	0.57
		BNT162b2	87.40 (82.77–91.17)	86.23 (79.34–91.50)	0.72
		Heterologous	71.14 (63.16–78.26)	86.50 (82.19–90.09)	0.58
	SD Biosensor (15%)	Total	88.81 (85.91–91.29)	78.71 (74.85–82.23)	0.68
		ChAdOx1	80.86 (74.86–85.96)	82.61 (77.37–87.07)	0.63
		BNT162b2	94.66 (91.20–97.05)	77.54 (69.66–84.20)	0.74
		Heterologous	76.67 (69.07–83.18)	83.92 (79.36–87.83)	0.59
	Roche (278.81 BAU/mL)	Total	70.52 (66.56–74.25)	91.57 (88.77–93.85)	0.61
		ChAdOx1	62.20 (55.25–68.80)	92.89 (88.99–95.73)	0.57
		BNT162b2	69.47 (63.50–74.98)	93.48 (87.98–96.97)	0.56
		Heterologous	58.00 (49.68–66.00)	94.86 (91.78–97.03)	0.58
	Abbott (57.48 BAU/mL)	Total	87.03 (83.97–89.70)	82.53 (78.90–85.76)	0.70
		ChAdOx1	72.25 (65.65–78.20)	87.35 (82.62–91.19)	0.60
		BNT162b2	95.42 (92.14–97.61)	83.33 (76.05–89.13)	0.80
		Heterologous	82.00 (74.90–87.79)	83.60 (79.01–87.54)	0.63

a Since the heterologous group was enrolled after the second sample collection of the ChAdOx1 and BNT162b2 groups, the results of the first and second sample collections of the ChAdOx1 group were imputed.

### Quantitative evaluation.

**FIG 2:**
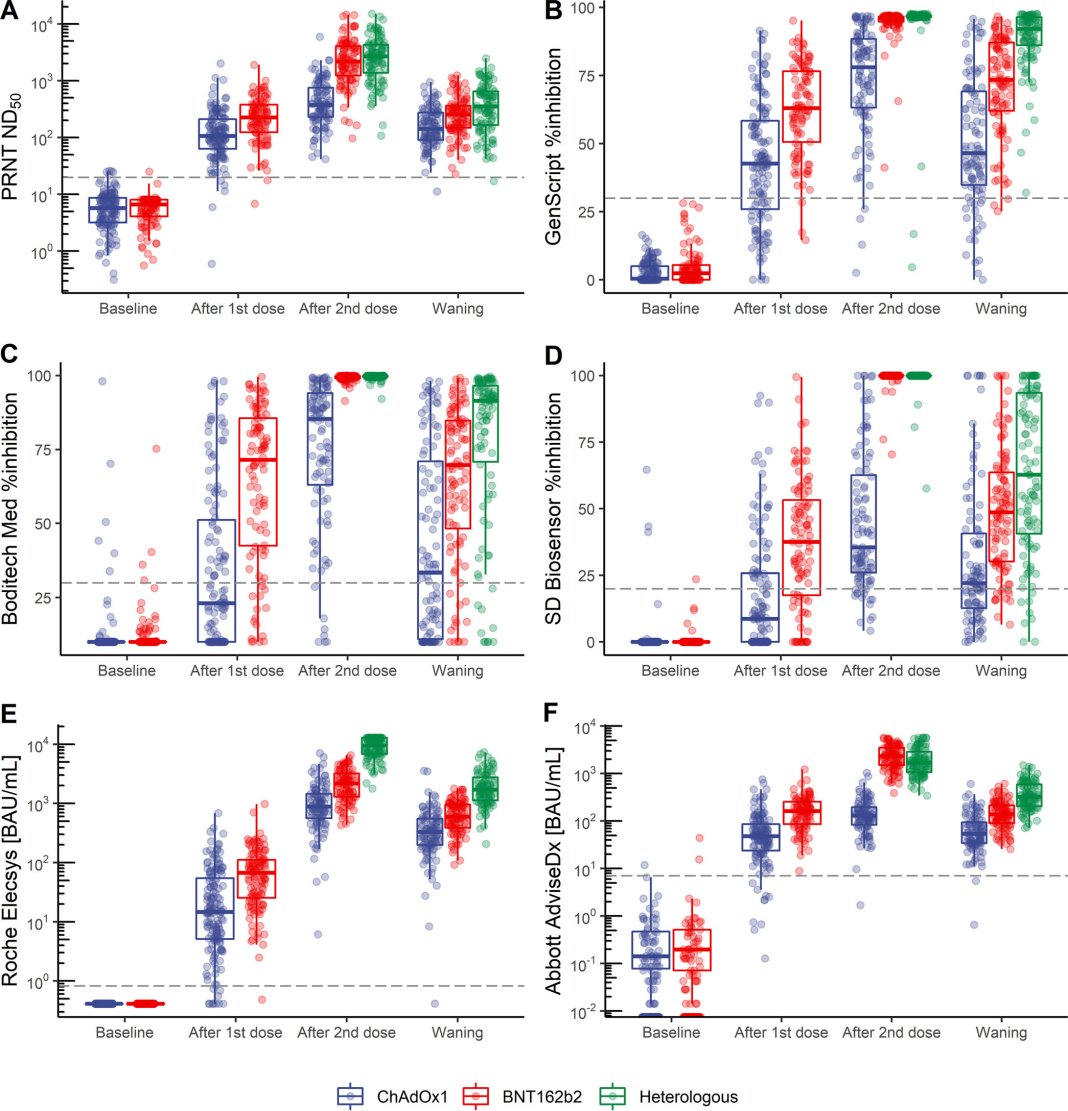
Box-and-whisker plots illustrating the results of each assay by vaccine group at different time points: (A) PRNT ND_50_, (B) GenScript, (C) Boditech Med, (D) SD Biosensor, (E) Roche, and (F) Abbott.

The quantitative results of each assay are depicted in Fig. 2 as dot plots overlaid on box-and-whisker plots at each time point according to the type of vaccination. Of the baseline samples, 6 (2.60%), 10 (4.33%), 4 (1.73%), and 3 (1.30%) showed positive results using the PRNT, BM, SD, and Abbott assays, respectively, but none using the GS and Roche assays. Compared with the PRNT, while the semiquantitative binding assays demonstrated a visually similar trend throughout the timeline, the sVNT assays exhibited a relatively narrow range of detection and early saturation at high titers. Although the first vaccination dose resulted in relatively low antibody titers, most samples collected after the first dose showed a PRNT ND_50_ value of ≥20, implying emerging neutralizing antibodies. Nonetheless, the sVNT assays, especially the BM and SD assays, were insufficient to suggest positive concordance with the PRNT, which was prominent in the ChAdOx1 group. The samples collected 2 weeks after the second dose in the BNT162b2 and heterologous groups showed nearly 100% inhibition using all three sVNT assays. During the waning period, the results showed a trend similar to those obtained after the first dose, where relatively low antibody titers resulted in false negativity using the BM and SD assays, particularly in the ChAdOx1 group. The BNT162b2 group showed a consistently higher neutralization titer than the ChAdOx1 group across all time points, and the heterologous group had the highest neutralizing activity during the waning period compared to the two homologous groups. See Table S1 in the supplemental material for detailed quantitative results of the assays at each time point and for each type of vaccination protocol.

**FIG 3:**
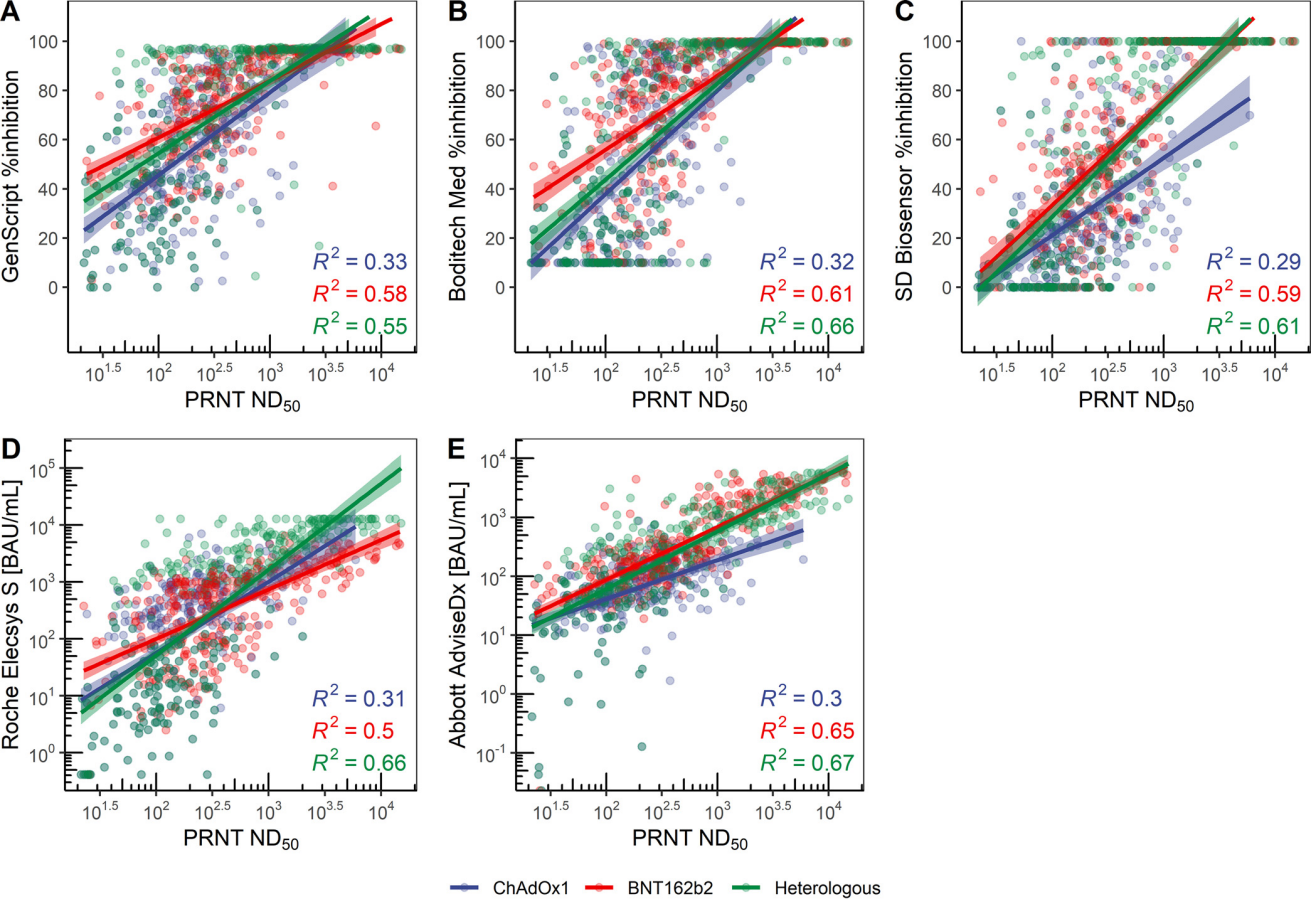
Quantitative correlation between PRNT ND_50_ and each assay: (A) GenScript, (B) Boditech Med, (C) SD Biosensor, (D) Roche, and (E) Abbott.

The quantitative results of each assay showed moderate to strong correlation with the titers of the PRNT in all vaccine groups, while the correlation coefficient (Spearman’s correlation) was lowest in the ChAdOx1 group. Overall, the coefficients of determination (*R*^2^) for the GS, BM, SD, Roche, and Abbott assays were 0.51, 0.58, 0.51, 0.49, and 0.56, respectively. The quantitative results of all assays showed strong correlation with the results of the log-converted PRNT. The results are illustrated in Fig. 3.

## DISCUSSION

In this study, we evaluated various assays in comparison with the PRNT to determine the feasibility of each assay for predicting neutralization activity. In terms of the qualitative performance of each assay in predicting a neutralizing PRNT ND_50_ value of ≥20 using the cutoff suggested by the manufacturer, the binding assays were superior to the sVNT assays. While the performance of the evaluated assays in predicting an ND_50_ value of ≥20 varied significantly depending on the vaccination protocol, the performance in predicting an ND_50_ value of ≥118.25 was comparable among the different assays using the optimal cutoffs determined for each assay.

Conventionally, an ND_50_ value of ≥20 is the cutoff used for determining the presence of neutralizing activity. However, this cutoff may not be a clinically appropriate guideline for immune protection *in vivo*, which requires a higher antibody titer. In addition, commercial assays and the cutoffs offered by the manufacturers are focused on ascertaining the presence of a certain amount of antibodies rather than determining a titer that represents a clinically protective effect. Therefore, we investigated the performance of each assay in predicting the protective effect, as well as new cutoffs for each assay that best represent a 50% protective effect (ND_50_, ≥118.25) derived from the convalescent-phase sera of 116 reverse transcription-PCR (RT-PCR)-confirmed patients.

The cutoffs for a protective effect obtained using Youden’s index were higher than the manufacturer cutoffs for all assays except the SD assay. These higher cutoff values provided higher agreement at the expense of sensitivity. Epidemiologically, false positivity rather than false negativity is a greater obstacle, since this could pose a risk to individuals without a sufficient protective effect. Thus, the use of these new cutoffs with higher specificity would be desirable. Regarding the SD assay resulting in an opposite trend with a lower cutoff for the protective effect, we believe that it was difficult to determine an appropriate cutoff because of the poor discriminative power at low antibody titers. However, with adjustment of the cutoff and a higher target titer indicating a protective effect, the performance of the assay was improved.

Although previous studies evaluating the performance of the SD sVNT assay in vaccinated individuals have demonstrated fair performance compared to the GS assay (12, 13), our study did not show comparable results. The BM and SD assays showed relatively poor performance due to poor sensitivity at low neutralizing antibody (nAb) titers, particularly after the first dose and waning point of the ChAdOx1 vaccine. Even though the semiquantitative binding assays from Roche and Abbott capture nonneutralizing binding antibodies as well, they resulted in a kappa agreement with a PRNT ND_50_ value of ≥20 that was greater than that of the sVNT assays. As binding antibodies against the RBD are not mutually exclusive with neutralizing antibodies but rather coexist with affinity and avidity maturation, the binding assays are also feasible for use in predicting the neutralizing effect, aside from the underlying assay mechanism. In addition, affinity maturation is achieved with the decay of antibodies with low affinity, which contributes to increased neutralization potency (14). Considering the recent rates of COVID-19 infection and vaccination, the majority of binding antibodies against the RBD would be neutralizing antibodies, which further implies the possibility of predicting the neutralization effect with semiquantitative binding assays. While it has been shown that semiquantitative binding assays are able to predict the neutralizing effect in patients naturally infected with COVID-19 (11), we suggest that this notion also applies to the vaccinated population.

Regarding the baseline samples with positive results in each assay, it has been shown that antibodies against coronaviruses other than SARS-CoV-2 could have cross-reactivity toward SARS-CoV-2 (15–17). Hence, when interpreting the positive results of baseline samples, the possibility of cross-reactivity should be considered. However, when the cutoffs indicating a protective effect were applied, only the BM and SD assays had false-positive results, which suggests a relatively poor performance of these two assays at low antibody titers. Moreover, as none of the samples that may have had cross-reactivity had a neutralizing effect sufficient for protective immunity, consistent with recent reports (15), this issue is not a major concern.

There have been multiple previous studies evaluating the performance of various sVNT assays and semiquantitative binding assays in relation to the PRNT (11, 18–24), whereas the ability to predict the protective immunity has not been evaluated to the best of our knowledge. Although the circulating antibody titers do not correlate with immune memory against SARS-CoV-2 (25), the neutralizing antibody titers were able to predict the protective immunity (26). While it is still uncertain whether the neutralizing antibodies mediate the protection *per se* or correlate with other immune responses (26), commercial antibody assays could predict immune protection in correlation with neutralizing activity regardless of the underlying machinery. Our study also has strengths in that it included a relatively large number of samples with a wide range of antibody titers. Moreover, this study included multiple time points and different vaccination protocols, which represent different antibody titers and antibody maturation characteristics.

A major caveat of this study is that it did not address issues related to SARS-CoV-2 variants of concern. A recent study suggested that further antibody maturation via a booster shot results in an increased neutralizing effect against variants of concern (27). Hence, even samples with comparable neutralizing titers against the wild type could possess different neutralizing activity against a certain variant of concern. Furthermore, neutralizing activity against variants of concern could largely vary for different strains. Different vaccines and prior SARS-CoV-2 infection with various strains further complicate this issue. Patients with natural infection originating from different variants of concern raise another concern, as antibodies produced by different variants could result in discordant performance of various assays. As new assays targeting different variants of concern are currently being developed, further evaluation regarding major strains, such as Omicron, is warranted. Another limitation is that samples taken after a booster shot or breakthrough infection were not included in the analysis. However, we believe that this is not a major concern for comparing the performance of different assays, as all assays were deemed to show fair performance at high titers. Rather, samples taken in the waning period with a prolonged interval since the last vaccination or after a breakthrough infection should be analyzed in further studies to determine the feasibility of using sVNT assays and semiquantitative binding assays for estimating the neutralizing activity, as antibody titers during this period are deemed to be low.

In this study, it was demonstrated that sVNT and semiquantitative binding assays are applicable for predicting the neutralization activity of vaccinated individuals by comparing the results of each assay with those of the PRNT. This study included three sVNT assays, two of which were novel sVNT assays developed in Korea. In addition, semiquantitative binding assays from Roche and Abbott that are popular and used globally were incorporated into the analysis. Regarding a higher titer that represents protective immunity, the present study demonstrated the need for a different cutoff for each assay that could improve the performance. Application of the newly determined cutoffs resulted in diminished interassay differences and comparable results among the evaluated assays, which conferred the feasibility of clinical usage.

## MATERIALS AND METHODS

### Clinical specimens.

A multicenter health care worker-based prospective cohort study of seroprevalence was undertaken in Korea, starting in March 2021 (8). Overall, 131 subjects in the homologous ChAdOx1 protocol group, 131 subjects in the homologous BNT162b2 protocol group, and 100 subjects in the heterologous protocol group, receiving a first dose of ChAdOx1 and second dose of BNT162b2 during the course of vaccination, were randomly selected (Table 3). Serially collected samples from the homologous groups (baseline, 3 weeks after the first dose, and 2 weeks and 5 months after the second dose) and heterologous group (2 weeks and 5 months after the second dose) were studied. The baseline samples were collected in the first 3 weeks of March 2021, and the first dose of the vaccine was administered in the second and third week of March 2021. The subjects were all recruited before July 2021, which was the beginning of dominance of the Delta variant in Korea (28).

**TABLE 3:** Baseline demographics

Characteristic	Data for:^*a*^			
	ChAdOx1	BNT162b2	Heterologous	Overall
Sex (no. [%])				
Male	38 (29.0)	32 (24.4)	18 (18.0)	88 (24.3)
Female	93 (71.0)	99 (75.6)	82 (82.0)	274 (75.7)
Age				
Median (yrs [IQR])^*b*^	37.0 (31.0–46.5)	34.0 (28.0–43.5)	37.0 (28.0–43.3)	36.0 (29.0–44.0)
<50 (no. [%])	105 (80.2)	121 (92.4)	91 (91.0)	317 (87.6)
≥50 (no. [%])	26 (19.8)	10 (7.6)	9 (9.0)	45 (12.4)

a ChAdOx1 group, *N* = 131; BNT162b2 group, *N* = 131; heterologous group, *N* = 100; total study population, *N* = 362.

b IQR, interquartile range.

### Assays.

Six different assays were utilized in this study: one PRNT, three sVNT assays, and two binding assays. The three sVNT assays used in this study were the cPass SARS-CoV-2 neutralization antibody detection kit (hereafter, GS), the AFIAS COVID-19 nAb kit (Boditech Med, Chuncheon, Gangwon-do, South Korea) (hereafter, BM), and the standard F SARS-CoV-2 nAb fluorescent immunoassay (FIA) (SD Biosensor, Suwon, Gyeonggi-do, South Korea) (hereafter, SD). The two semiquantitative binding assays utilized in this study were the Elecsys anti-SARS-CoV-2 S kit (Roche Diagnostics, Rotkreuz, Switzerland) (hereafter, Roche) and the AdviseDx SARS-CoV-2 IgG II Quant kit (Abbott Laboratories, Abbott Park, IL, USA) (hereafter, Abbott). The PRNT using wild-type SARS-CoV-2 was carried out by the Korea Disease Control and Prevention Agency, and the detailed procedure for the PRNT is described in our previous publication (8, 11). The PRNT was considered positive for a 50% neutralizing dose (ND_50_) of ≥20. Other commercial sVNT and binding assays were conducted following the manufacturer’s instructions at Samsung Medical Center, Seoul, South Korea. The cutoffs of the GS, BM, SD, Roche, and Abbott assays suggested by the manufacturers were ≥30%, ≥30%, ≥20%, 0.82 BAU/mL, and 7.1 BAU/mL, respectively.

### Statistical analysis.

The sensitivity, specificity, and Cohen’s kappa were calculated to compare the performance among different assays. Cohen’s kappa was interpreted using the following criteria: values of <0.00 were considered to be in poor agreement, 0.00 to 0.20 in slight agreement, 0.21 to 0.40 in fair agreement, 0.41 to 0.60 in moderate agreement, 0.61 to 0.80 in substantial agreement, and 0.81 to 1.00 in almost perfect agreement (29). Moreover, in addition to the performance in predicting a PRNT ND_50_ value of ≥20, the performance of each assay in predicting an ND_50_ value of ≥118.25 was assessed, since it was the cutoff representing the 50% protective effect. The PRNT ND_50_ value indicating 50% protective effect was determined using the methods of Khoury et al. (26) using 188 sera from 116 RT-PCR-confirmed patients, collected after 28 days of illness, with a median PRNT result of 245 (interquartile range [IQR], 57 to 1,168) (11). As the manufacturer cutoffs were designed for the detection of a certain level of antibodies rather than to ascertain the protective effect, new cutoffs for each assay that best predicted the protective effect were obtained using Youden’s index [sensitivity + (1 − specificity)]. All statistical values were calculated using R 4.0.2 (R Foundation for Statistical Computing, Vienna, Austria) (30), and the plots were illustrated using the ggplot2 3.3.2 (31) package in R 4.0.2 (30).

### Ethics statement.

The study was approved by the institutional review board (IRB) of each hospital, and written informed consent was obtained from each participant. The hospitals are as follows: Samsung Medical Center, Gangman Severance Hospital, Yongin Severance Hospital, Severance Hospital, Seoul National University Bundang Hospital, Asan Medical Center, Chungbuk National University Hospital, Kyungpook National University Hospital, Kyungpook National University Chilgok Hospital, Korea University Ansan Hospital.
