# Supplementary material for: Estimation of SARS-CoV-2 Neutralizing Activity and Protective Immunity in Different Vaccine Types Using Three Surrogate Virus Neutralization Test Assays and Two Semiquantitative Binding Assays Targeting the Receptor-Binding Domain
Source: Microbiol Spectr. 2022 Oct 17;10(6):e02669-22. doi: 10.1128/spectrum.02669-22 (PMC9769750; doi:10.1128/spectrum.02669-22)
Supplement: Supplemental file 1 — Supplemental material. Download spectrum.02669-22-s0001.pdf, PDF file, 0.2 MB [file spectrum.02669-22-s0001.pdf]

Supplementary Materials

List of Contents

Procedures of sVNT assays and binding assays

Supplemental Table 1. Quantitative results of the evaluated assays at each timepoint depending on the type of vaccination protocol

Procedures of sVNT assays and binding assays

sVNT assays

Three different sVNT assays were utilized in this study: 1) cPass SARS-CoV-2 Neutralization Antibody Detection Kit (GenScript, Piscataway, NJ, USA), 2) AFIAS COVID-19 nAb (Boditech Med, Chuncheon, Gangwon-do, Korea), and 3) Standard F SARS-CoV-2 nAb FIA (SD Biosensor, Suwon, Gyeonggi-do, Korea). All assays determine the degree of interaction between the SARS-CoV-2 receptor binding domain (RBD) of spike protein and the recombinant human angiotensin converting enzyme 2 (hACE2) receptor. The interaction between the RBD and hACE2 is inhibited by neutralizing antibodies binding to the RBD. The cPass SARS-CoV-2 Neutralization Antibody Detection Kit (GenScript) is an enzyme-linked immunosorbent assay (ELISA), which measures the optical density (absorbance at 450 nm) to calculate the inhibition ratio. On the other hand, the AFIAS COVID-19 nAb (Boditech Med) and the Standard F SARS-CoV-2 nAb FIA (SD Biosensor) are a fluorescence immunoassay (FIA), measuring the fluorescence intensity to calculate the inhibition ratio.

Binding assays

Two different binding assays were utilized in this study: 1) Elecsys Anti-SARS-CoV-2 S kit (Roche Diagnostics, Rotkreuz, Switzerlande) and 2) AdviseDx SARS-CoV-2 IgG II Quant kit (Abbott Laboratories, Abbott Park, IL, USA). The Elecsys Anti-SARS-CoV-2 S kit (Roche) is an electro-chemiluminiscence immunoassay (ECLIA), capturing the binding antibodies by a double-anitgen sandwich principle using recombinant RBD. The kit captures IgG antibodies predominantly but is also capable of detecting IgA and IgM. The AdviseDx SARS-CoV-2 IgG II Quant kit (Abbott

Laboratories) is a chemiluminescent microparticle immunoassay (CMIA), measuring IgG binding antibodies with recombinant RBD.

Supplemental Table 1. Quantitative results of the evaluated assays at each timepoint depending on the type of vaccination protocol

**Assay, Unit**	**Vaccine type**	**Assay results***			
		**Baseline**	**After 1st dose**	**After 2nd dose**	**Waning**
**PRNT, ND_{50}**	**ChAdOx1**	5.72 (3.17–8.66)	106.59 (63.29–210.27)	374.12 (230.07–750.27)	141.09 (90.51–275.19)
	**BNT162b2**	6.61 (4.08–8.00)	225.51 (123.16–376.66)	2181.96 (1230.25–4046.88)	254.81 (148.68–360.25)
	**Heterologous**	NA	NA	2688.71 (1377.85–4284.23)	355.13 (165.87–648.54)
**GenScript, %**	**ChAdOx1**	0.48 (0.00–4.97)	42.7 (25.96–58.4)	78.12 (63.24–88.44)	46.52 (34.83–69.16)
	**BNT162b2**	2.39 (0.00–5.43)	63.08 (50.56–76.62)	96.18 (94.78–96.66)	73.4 (62.06–87.17)
	**Heterologous**	NA	NA	96.79 (96.62–97.01)	92.14 (86.15–96.44)
**Boditech Med, %**	**ChAdOx1**	10.00 (10.00–10.00)	23.1 (10.00–51.17)	85.41 (63.04–94.11)	33.45 (10.9–70.98)
	**BNT162b2**	10.00 (10.00–10.10)	71.55 (42.43–85.58)	99.48 (99.05–99.81)	69.75 (48.25–84.75)
	**Heterologous**	NA	NA	99.80 (99.60–99.90)	91.50 (70.88–96.52)
**SD Biosensor, %**	**ChAdOx1**	0.00 (0.00–0.00)	8.70 (0.00–25.75)	35.45 (26.00–62.60)	22.15 (12.68–40.62)
	**BNT162b2**	0.00 (0.00–0.00)	37.55 (17.58–53.25)	100.00 (100.00–100.00)	48.65 (30.08–63.57)
	**Heterologous**	NA	NA	100.00 (100.00–100.00)	62.80 (40.55–93.50)
**Roche, BAU/mL**	**ChAdOx1**	0.41 (0.41–0.41)	14.71 (5.11–54.32)	889.92 (562.76–1450.62)	331.28 (197.79–552.73)
	**BNT162b2**	0.41 (0.41–0.41)	67.49 (25.41–111.11)	2165.64 (1288.84–3197.02)	595.68 (388.89–956.28)
	**Heterologous**	NA	NA	9532.92 (6852.11–12860.08)	1712.96 (1119.86–2752.06)
**Abbott, BAU/mL**	**ChAdOx1**	0.01 (0.00–0.16)	48.32 (23.96–85.73)	130.37 (83.73–196.42)	54.56 (34.10–93.79)
	**BNT162b2**	0.04 (0.00–0.20)	161.93 (85.90–254.21)	2335.02 (1504.25–3518.36)	139.27 (89.58–213.61)
	**Heterologous**	NA	NA	1704.58 (1070.72–2886.8)	324.58 (204.96–593.16)

*Assay results are presented as the median (IQR). NA, not applicable.
